# Blood Pressure measurements are site dependent in a cohort of patients with neurological illness

**DOI:** 10.1038/s41598-020-60414-7

**Published:** 2020-02-25

**Authors:** Kathrina B. Siaron, Michaela X. Cortes, Sonja E. Stutzman, Aardhra Venkatachalam, Khalid Mohamed Ahmed, DaiWai M. Olson

**Affiliations:** University of Texas Southwestern Medical Center, Dallas, TX USA

**Keywords:** Health care, Neurology

## Abstract

Blood pressure (BP) management is a crucial part of critical care that directly affects morbidity and mortality. While BP has become a mainstay in patient care, the accuracy and precision of BP measures across commonly used sites (left upper arm, right upper arm, etc.) and methods have not been established. This study begins to fill this gap in literature by testing the null hypothesis that BP measurement does not vary according to site. This is a prospective, non-randomized, cross-sectional study of 80 neurocritical care unit patients. Near simultaneous non-invasive blood pressure (NIBP) readings from 4 different locations (bilateral upper arm, bilateral wrist) and, when available, intra-arterial blood pressure readings (IABP) were included. Pearson correlation coefficients and one-way repeated measures ANOVA were used to observe the systolic, diastolic, and mean arterial pressure (MAP) correlations. The BP measured at the four most common sites (left upper arm, left wrist, right upper arm, right wrist) had adequate correlation coefficients but were statistically significantly different and highly unpredictable. The median inter-site systolic variability was 10 mmHg (IQR 2 to 10 mmHg). The median inter-site MAP variability was 6mmHg with an interquartile range (IQR) of 3 to 9 mmHg. As expected, the values correlated to show that patients with high BP in one site tended to have high BP in another site. However, the unpredictable inter-site variability is concerning within the clinical setting where oftentimes BP measurement site is not standardized but resulting values are nevertheless used for treatment. There is prominent inter-site variability of BP measured across the 4 most common measurement sites. The variability persists across non-invasive (NIBP) and invasive (IABP) methods of assessment.

## Introduction

Blood pressure (BP) monitoring is essential to neuroscience intensive care unit (NSICU) patient management. Adequate cerebral perfusion is dependent upon central BP control, maintained through the modulation of various factors such as cardiac output, vascular tone, systemic vascular resistance, and intravascular volume. Although BP can be measured at multiple sites (e.g., upper arm, wrist, thigh), there is no gold standard for site selection. Inconsistencies in BP readings may occur due to inter-observer variability, technical issues, or methods and locations for measurement. The true value of a patient’s BP cannot be measured with total precision, owing to its natural variability over time, as well as the limitations of the methods used to measure BP^1^. Moreover, BP measurement is influenced by other physiological factors, such as position changes (e.g., orthostatic stress due to gravitational blood volume shift) and external factors, such as the measurement site used for the non-invasive blood pressure (NIBP) machine^2^. This variability lends ambiguity to BP measurements in the critical care setting, where accuracy is paramount as a basis for medical interventions. It is particularly crucial in the NSICU setting, where minute fluctuations in Intracranial Pressure (ICP) and Cerebral Perfusion Pressure (CPP) can lead to acute deterioration of the patient’s neurological status^3^.

To better understand BP management in the setting of neurological diseases such as tumors and stroke, it is important to have a firm grasp of cerebrovascular physiology and its compensatory mechanisms at the onset of acute injury. Under normal conditions, the brain adjusts the downstream cerebrovascular resistance by reflex vasoconstriction or vasodilation of cerebral arterioles, a function known as autoregulation. This ensures that cerebral blood flow remains relatively constant across a wide range of mean arterial pressure (MAP)^3^. Evidence has shown that cerebral autoregulation becomes dysfunctional after injuries such as an acute stroke, and cerebral blood flow becomes more susceptible to fluctuations in MAP, potentially exposing brain tissue to hypoperfusion and subsequent ischemia or hypertensive damage and possible hemorrhage^3^. Overall, BP is also transiently elevated in the setting of acute stroke due to neuroendocrine factors, physical stress, infarct topography, and central mechanisms of BP control^4^.

Invasive arterial blood pressure (IABP) measurement is considered to be the gold standard of BP measurement in the critical care setting^5^ as it is able to measure the beat-to-beat value of BP in the absence of technical errors, reflecting the constant fluctuations of BP in real time. There are, however, various technical limitations associated with the method, such as: errors due to over- or under-dampening, calibration errors, motion artifacts, in-line clotting, or the presence of pre-existing systemic vascular disorders affecting the integrity of the patient’s arteries. The IABP measurement, maintenance, and troubleshooting requires more clinical expertise for safe management. Additionally, it is an invasive device that carries its own host of risks. It is therefore common practice to use NIBP machines for majority of patients within and beyond critical care, as well as during the hyperacute phase of managing critical emergencies when an arterial access has not yet been established^6^. The substitution of NIBP for IABP measurement is done despite the limited amount of data available concerning their correlation; furthermore, the impact of age, sex, and body mass index (BMI) on NIBP-IABP correlation has not been studied^7^. This study aims to address the gap by observing the correlations between two different methods of BP measurement (NIBP and IABP) used in the NSICU and across four different measurement sites (bilateral upper arms and bilateral wrists).

## Methods

This is a prospective, non-randomized, observational study to correlate blood pressure readings in acutely ill patients admitted with a neurological diagnosis to a 20-bed NSICU in a comprehensive stroke certified academic center. The team collected data from a total of 82 patients over a period of four months (April-July 2019). All study procedures were approved by the University of Texas Southwestern Medical Center Institutional Review Board prior to the initiation of the study. Likewise, the study team weighed the risks and benefits to the patients before initiation and ensured that all study procedures followed Helsinki guidelines. Patients were screened daily using the electronic medical record (EMR). Patients were eligible for the study if they were: over 18, were English speaking, and were admitted to the NSICU with a neurological diagnosis. Patients were excluded if they had any of the following: contra-indication to bilateral upper arm or wrist NIBP measurements, missing an upper limb or upper limb fracture, or a diagnosis of Raynaud’s disease. Once deemed eligible, the patient or their legally authorized representative were consented for the study through a written informed consent.

After consent was obtained, we consulted with the patient’s primary nurse to ensure that the patient was in an appropriate condition to perform measurements. A stopping rule was that if the patient was clinically unstable, unable to tolerate multiple NIBP measurements for any reason (i.e. pain, emotional distress), unable to tolerate having the head of bed at 30 degrees, or otherwise indisposed, we would terminate the attempt and return at another time. No patients met this stopping rule and all measurements were completed during the first encounter.

**Figure 1:**
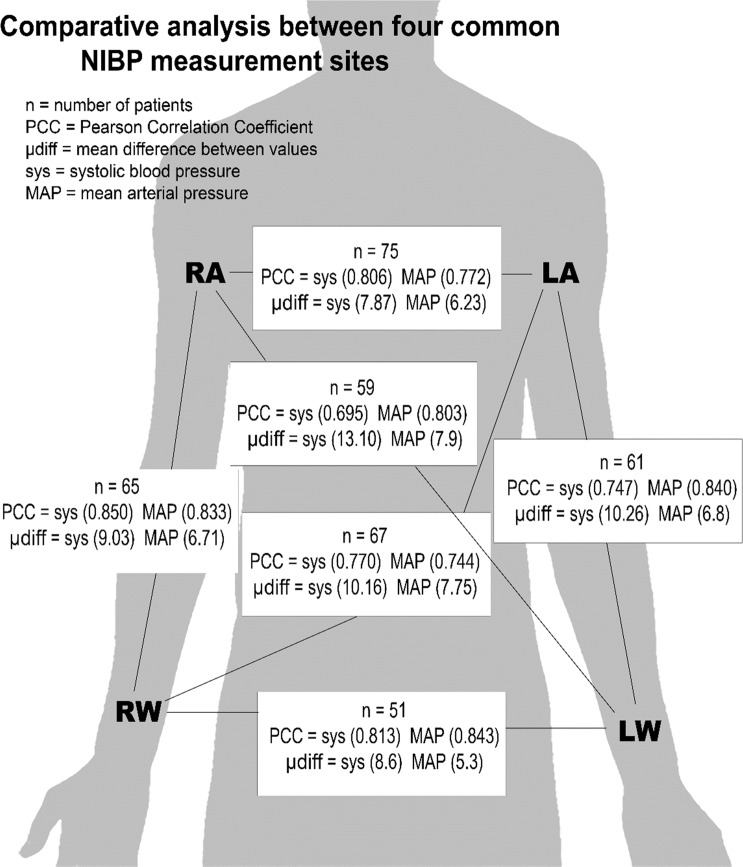
Comparative analysis between four common NIBP measurement sites.

Each enrolled patient underwent a one-time BP reading from four different measurement sites at near-simultaneous times. We identified and placed appropriately-sized BP cuffs on the left upper arm (LA), right upper arm (RA), right wrist (RW), and/or left wrist (LW) (Fig. 1) for NIBP measurements, using manufacturer-designated wrist cuffs for the wrist NIBPs. Wrist NIBP sites are not routinely used in this ICU except during difficult clinical settings when other measurement sites are not available, but for the sake of inter-site comparison, the measurements were included. Patients were appropriately preconditioned for NIBP measurement: measurements were taken on the bare limb, free of thick cloth. The patient was placed in the upright position with the head of the bed at 30 degrees for the duration of the measurement. If an arterial line was present, the IABP measurement was included in lieu of one of the wrist NIBP measurements, with an arterial line reading recorded simultaneous to the first NIBP measurement result.

All blood pressure values were obtained using the IntelliVue MP40 (Philips North America Corp. Andover, MA) monitoring system. To obtain near-simultaneous readings, researchers used two separate IntelliVue monitors that provided oscillometric NIBP readings. If an arterial line was present, the transducer was calibrated and leveled by the primary nurse prior to the reading, ensuring a normal flush test, adequate dampening, and brisk aspiration. The IABP value was noted simultaneous to the first NIBP reading. The research team member noted the location of the arterial line (i.e., right or left radial, brachial, or femoral) as well as the patient’s heart rate and cardiac rhythm at the time of the first NIBP reading. The first measures were taken in the upper arms, with the study member pushing the “Start” button on the two monitors simultaneously. Immediately after the BP values appeared on the IntelliVue monitors, a reading was taken from the arterial line (if present). Then the two monitors were connected to the wrist cuffs and BP measurements were taken in the lower arms. The BP measurements were first recorded with paper and pen. After sampling, all demographic data and BP measurements were abstracted to an electronic spreadsheet before being converted to SAS v 9.4 (SAS Institute) for analysis.

### Statistical analysis

Sample size was estimated using data from McNett *et al*.^8^ From 136 patients with paired samples, there was a statistically significant difference of 9 mm Hg in SBP using head of the bed (HOB) location (141.63 [25.229] versus 129.40 [28.704]). From these data, it was estimated that 78 patients were adequate to reject the null hypothesis (effect size = 0.45, power = 0.80, and a two-tailed alpha = 0.05)^9^. This number was rounded upwards to remain conservative, and a sample of 80 was selected.

Descriptive data were derived for BP from each site (e.g. LA, RA, LW, RW, arterial line). After the initial assumption of approximately normal distribution was rejected for 3 BP measures (Shapiro-Wilk p < 0.05), all BP measures were log transformed (Shapiro-Wilk range p = 0.17 – p = 0.88). Histograms were constructed to test the assumption that blood pressure values are approximately normally distributed. An ANOVA test was used to identify differences in means of blood pressure by site. Simple regression models were used to examine association of blood pressure with the reference site (LA) and other sites. Multivariate regression was used to explore any potential moderator effect.

## Results

Of 82 patients enrolled, 2 were excluded as being previously enrolled (1) on a prior admission and 1 withdrew prior to BP sampling. Of 80 patients in this analysis, 41(51.3%) were male, mean age = 52.84 +/− 17.94) years old, and mean BMI = 30.18 +/− 7.74). Demographics were representative of the local population, majority Caucasian and non-Hispanic as detailed in Table 1. The top 5 admission diagnosis included ischemic stroke (12), subarachnoid hemorrhage (11), neoplasm (17), status epilepticus (7), and unruptured aneurysm or arteriovenous fistula/malformation (7); the remaining patients represented various diagnoses common to NSICUs. Hypertension and hyperlipidemia were the most common pre-existing history amongst the enrolled patients. Only 3 patients were receiving intravenous vasopressors at the time of enrollment. Notably, 32 (40%) enrolled were non-surgical; 14 (17.5%) underwent craniotomy for tumor resection, aneurysm clipping, or hematoma evacuation; and 8 (10%) underwent stenting and/or coiling of an aneurysm. Six patients (7.5%) underwent thrombectomy after an ischemic stroke, and two patients (2.5%) had extraventricular drains (EVDs) in place while in the NSICU.

**Table 1:** Demographics and characteristics of enrolled patients.

Variables	n (%)
**Demographics**	
Caucasian	50 (70.0%)
African-American	17 (21.3%)
Asian	4 (4.5%)
Other	3 (3.8%)
Hispanic	69 (86.3%)
Non-Hispanic	11 (13.8%)
**Medical History**	
Hypertension	39 (48.8%)
Hyperlipidemia	25 (31.5%)
Diabetes, type I or II	20 (25.0%)
Stroke	14 (17.5%)
Smoker, past or active	13 (16.3%)
Atrial fibrillation	10 (12.5%)
Anxiety	5 (6.3%)
**Diagnosis**	
Tumor resection	17 (21.3%)
Ischemic stroke	11 (13.8%)
Subarachnoid hemorrhage	11 (13.8%)
Seizures	7 (8.8%)
Aneurysm (unruptured)	4 (5.0%)
Arteriovenous malformations	3 (3.8%)
Encephalitis	1 (1.3%)
Other	26 (32.5%)

Both left and right mean upper arm NIBP [systolic/diastolic (MAP)] were lower than left and right mean wrist NIBP [LA 125/71 (86); RA 124/71 (85); LW 130/76 (90); RW 128/73 (88); p < 0.0001]. Of the 29 patients with arterial access, 16 had left radial arterial lines [mean IABP = 127/61 (80)], 7 had right radial arterial lines [mean IABP = 139/63 (88)], 4 had left brachial arterial lines [mean IABP = 138/73 (91)], and 2 had right brachial arterial lines [mean IABP = 122/68 (85)].

Systolic readings were isolated and comparative analysis of two different measurement sites showed significant differences in blood pressures (Fig. 1). The LA and RA, the two most common measurement sites for NIBP, notably showed a mean difference of 7.87 +/−7.23 mm Hg with a total range of 0 to 30 mm Hg. The largest mean difference in systolic pressures was between LW and RA (13.10 +/− 10.19; range = 0 to 57 mm Hg). The mean difference in LA and RA diastolic readings was 6.33 +/− 5.58 (range = 0 to 25 mm Hg). The largest difference mean difference in diastolic pressures was between LW and RA (7.61 +/− 5.80; range = 0 to 31 mm Hg). The mean difference in LA and RA MAP readings was 6.23 +/− 5.80 (range = 0 to 27 mm Hg). The largest difference mean difference in MAP pressures was between LW and RA (7.93 +/− 6.23; range = 0 to 36 mm Hg). For further statistical clarity and analysis, Table 2 shows the absolute mean differences & true mean differences compared across measurement sites for systolic, diastolic, and MAP.

**Table 2:** Inter-site comparison of absolute differences & true differences in mmHg.

Variable	n		Absolute Difference		True Difference	
		Mean (SD)		Range (min-max)	Mean (SD)	Range (min-max)
Left arm	Systolic	75	7.87 (7.23)	30 (0–30)	0.61 (10.71)	56 (−30–26)
vs	Diastolic		6.33 (5.58)	25 (0–25)	0.76 (8.44)	40 (−15–25)
Right arm	MAP		6.23 (5.80)	27 (0–27)	0.89 (8.49)	48 (−21–27)
Left arm	Systolic	67	10.16 (7.07)	32 (1–33)	−1.45 (12.36)	53 (−20–33)
vs	Diastolic		7.18 (6.18)	26 (0–26)	−1.93 (9.31)	47 (−24–21)
Right wrist	MAP		7.75 (6.21)	24 (0–24)	−1.84 (9.80)	47 (−24–23)
Left arm	Systolic	61	10.26 (10.08)	51 (0–51)	−5.41 (13.37)	77 (−51–26)
vs	Diastolic		7.13 (5.08)	28 (0–28)	−3.59 (8.03)	43 (−28–15)
Left wrist	MAP		6.80 (5.85)	32 (0–32)	−4.02 (8.06)	48 (−32–16)
Left wrist	Systolic	59	13.10 (10.19)	57 (0–57)	6.42 (15.38)	81 (−24–57)
vs	Diastolic		7.61 (5.80)	31 (0–31)	4.73 (8.36)	45 (−14–31)
Right arm	MAP		7.93 (6.23)	36 (0–36)	5.25 (8.65)	48 (−12–36)
Left wrist	Systolic	51	8.59 (7.66)	34 (0–34)	1.22 (1.50)	49 (−15–34)
vs	Diastolic		5.96 (5.69)	23 (1–24)	1.10 (8.21)	45 (−24–21)
Right wrist	MAP		5.33 (4.80)	19 (0–19)	7.19 (0.00)	35 (−19–16)
Right wrist	Systolic	65	9.03 (6.48)	26 (0–26)	2.72 (10.83)	49 (−26–23)
vs	Diastolic		7.06 (6.83)	35 (0–35)	2.57 (9.52)	56 (−21–35)
Right arm	MAP		6.71 (5.82)	31 (0–31)	3.11 (8.35)	50 (−19–31)

Pearson Correlation Coefficient (PCC) matrix was used to examine NIBP in LA, RA, LW, RW, and IABP for systolic, for diastolic, and for MAP (Table 3; Fig. 1). PCCs for systolic ranged from 0.04 (LW vs IABP) to 0.85 (RW vs RA). PCCs for diastolic ranged from 0.36 (RW vs IABP) to 0.83 (LW vs LA). PCCs for MAP ranged from 0.38 (LW vs IABP) to 0.84 (RW vs LW). Next, one-way repeated measures ANOVA models were constructed to examine within-subject NIPB variations across LA, RA, LW, and RW (n = 47). There was a statistically significant difference in within-subject systolic NIBP measurements (p = 0.03), diastolic NIBP measurements (p = 0.0002), and MAP measurements (p = 0.0001).

**Table 3:** Pearson Correlation Coefficient Matrix for inter-site comparison.

INTER-SITE COMPARISON PCC (p-value)	SYSTOLIC	DIASTOLIC	MEAN ARTERIAL PRESSURE (MAP)
Left Arm – Right Arm *(n* = *75)*	0.806 (<0.0001)	0.774 (<0.0001)	0.772 (<0.0001)
Left Arm – Left Wrist *(n* = *61)*	0.747 (<0.0001)	0.832 (<0.0001)	0.840 (<0.0001)
Left Arm – Right Wrist (*n* = *67)*	0.770 (<0.0001)	0.750 (<0.0001)	0.744 (<0.0001)
Right Arm – Right Wrist *(n* = *65)*	0.850 (<0.0001)	0.747 (<0.0001)	0.833 (<0.0001)
Right Arm – Left Wrist *(n* = *59)*	0.695 (<0.0001)	0.806 (<0.0001)	0.803 (<0.0001)
Right Wrist – Left Wrist *(n* = *51)*	0.813 (<0.0001)	0.779 (<0.0001)	0.843 (<0.0001)
Arterial – Left Arm *(n* = *28)*	0.755 (<0.0001)	0.500 (0.0042)	0.662 (0.0001)
Arterial – Right Arm *(n* = *25)*	0.812 (<0.0001)	0.642 (0.0007)	0.798 (<0.0001)
Arterial – Left Wrist *(n* = *12)*	−0.045 (0.8895)	0.544 (0.0895)	0.381 (0.2223)
Arterial – Right Wrist *(n* = *20)*	0.853 (<0.0001)	0.359 (0.1235)	0.626 (0.0031)

## Discussion

Our data showed large values for mean differences for commonly used measurement sites, indicating that blood pressure is not the same throughout the body at any given time; moreover, this indicates that one measurement site’s result cannot be used to substitute or infer a different site’s result. Our findings support and extend those of Vinyoles *et al*.^10^ who found a lack of concordance in left and right upper arm BP measurements. In simplest terms, when a clinician measures the blood pressure on the left arm, the clinician only has the value for the left arm. It is worth noting that some inter-site mean differences were above 10 mmHg (LA-RW, LA-LW, and LW-RA) and all inter-site mean difference comparisons were at *least* 7 mmHg for SBP, which is often the treatment parameter used for patients in the NSICU (Table 2). When distribution was analyzed using interquartile range, the range of mean differences between SBP readings were all 10 to 11 mmHg apart. This is significant since even 5 mmHg on the SBP is enough to make a difference when deciding to intervene or withhold treatment.

Mean differences between inter-site MAP comparisons followed the same trend, but the values were smaller than those for SBP. Interquartile range distribution showed that the range of mean differences between MAP readings were all 6 to 7 mmHg apart. Although mean pressure is the true driving pressure for peripheral blood flow and MAP is the parameter physically measured by oscillometric devices, current guidelines for clinical practice have been slow to incorporate NIBP MAP for vital signs monitoring and decision-making in the NSICU^11^. This is a point of concern since SBP targets are recommended in American Heart Association (AHA) guidelines where cerebral perfusion is at risk even though CPP, which calculates from the MAP, appears to be a more appropriate target. There are currently no established guidelines for MAP or CPP measurement in neurocritical care^7^. Given the wider mean differences between SBP readings from two sites that also reflect strong correlation coefficients, MAP appears to be the more consistent metric by which to decide treatment in the clinical setting. However, it is still important to consider that the MAP will not be exactly the same at any given time from site to site.

Upper arm measurements were consistently lower than wrist measurements, which could be attributed to the anatomical differences between the upper arm and the wrist, insofar that the wrist arteries are surrounded by tendons and bones and therefore less reactive to the external pressure applied by the NIBP cuff. The wrist sites are also more distal to the heart, and a more proximal site such as the upper arm would reflect central pressures more accurately. This consistent mismatch is worth considering in the clinical setting and in practice, we suggest avoiding wrist NIBPs if at all possible and other alternatives are available.

The AHA guidelines for the management of intracerebral hemorrhage^12^, unruptured intracranial aneurysm^13^, and acute ischemic stroke^14^ do recommend strict BP control but they do not specify a measurement site. The AHA guidelines for the measurement of BP in humans recommends that NIBP should be measured in the upper arm but it does not specify which arm; it does recommend using the higher BP reading for treatment purposes in the case of clinically significant inter-arm differences (defined as SBP or DBP difference ≥10 mmHg)^15^. However, this recommendation is for the management of hypertension in the general population and does not account for the special needs of neurocritical care patients, especially as some NSICU cases require permissive hypertension for cerebral perfusion (such as in acute ischemic stroke or high-grade vessel stenosis). A small correlational study^4^ conducted in 2007 observed that: (1) systolic NIBP readings consistently underestimate the systolic IABP readings by a significant margin, and (2) the higher the patient’s SBP, the greater the discrepancy between systolic IABP and NIBP. This makes the AHA recommendations even more problematic, as there is no existing guideline for when a clinician must decide between correct but significantly different NIBP and IABP measurements for treatment.

Although there was a smaller number of enrolled patients with arterial lines, it shows that IABP readings demonstrate greater range, variance, and weaker inter-site correlation than the NIBP readings. This is an important point of consideration in the NSICU setting, where IABP is preferred over NIBP when deciding on treatment. This correlational unreliability effectively challenges the widely accepted concept that IABP is the “gold standard” for BP measurement. Notably, applying a Bonferroni correction to the Pearson Correlation Coefficients resets the rejection for the null to p < 0.0017 and two of the correlations in Table 3 would fail to be rejected (both are correlations against the IABP). Further comparative research is warranted to better understand the correlation and inter-reliability between IABP and NIBP, especially as most existing research previously done on ICH and BP control used NIBP (including INTERACT 1 & 2)^16,17^.

Limitations for this study include its relatively small sample size enrolled from a single center. The study was designed for pragmatic (convenience, non-probability, non-randomized) sampling because the nurse investigators could not work 7-days a week; however, this sampling strategy has been successfully used in clinical nursing research^18^. It should also be noted that we used the first and only NIBP measure for each site, whereas guidelines recommend taking multiple measurements. However, our design reflects the practice used in most intensive care units wherein the NIBP is checked once unless the value is considered the product of an obvious measurement error. Some measurements were performed on stroke patients’ paretic arm, which can alter the NIBP readings due to differences in muscle tone^19^.

The use of nearly-simultaneous measurements was pragmatic and it is possible that hemodynamic changes are responsible for all of the BP differences; this could be examined in a future study. Due to the limitations of current NIBP technology, we are unable to ensure (even if the measurements were initiated at the exact same time on both arms) that the machine will give a result at the same time, hence the study was limited to near-simultaneous results. Additionally, it is not possible to retrieve wrist and upper arm results from the same arm at the same time due to the mechanics of NIBP measurement restricting blood flow to the distal extremity.

Furthermore, this study is not designed to be able to determine which measurement site or method is best used as a standard of practice. It is worth note that in patients who suffer rapid BP fluctuations (such as those with atrial fibrillation or other cardiac dysrhythmias), NIBP machines are limited in their capacity to track real-time BP changes. Although it was not an exclusion criterion, none of the enrolled patients in this study had atrial fibrillation or atrioventricular blocks.

Finally, this study was not structured to test the hypothesis on healthy participants, which could serve to highlight the hemodynamic changes brought about by neurovascular diseases and also verify whether or not the inter-site variabilities we found in neurologically ill patients remain consistent even in a healthy cohort. Future research using different methodological approaches to explore this old issue may yield different results.

## Conclusion

The study highlights the inter-site variability of BP using both NIBP and IABP measurements. Comparative analysis showed positive correlation between different measurement sites, but the difference in BP values was neither fixed nor predictable in any two particular sites. Each measurement site yielded distinctly different results from the others. Therefore, location *does* matter when measuring BP; however, this study cannot conclusively recommend which site to use above others. Further investigation through larger trials is warranted to better understand the correlation between different measurement sites and methods, as well as to identify the most accurate method of measuring BP in the clinical setting. In light of these findings, clinicians should consider standardizing the BP measurement site and method upon admission for consistent readings and better clinical decision-making.
